# A Comprehensive Prospective Comparison of Acute Skin Toxicity after Hypofractionated and Normofractionated Radiation Therapy in Breast Cancer

**DOI:** 10.3390/cancers13225826

**Published:** 2021-11-20

**Authors:** Kai J. Borm, Johanne Kleine Vennekate, Jan Vagedes, Mohammad O. A. Islam, Marciana N. Duma, Maximilian Loos, Stephanie E. Combs, Kilian Schiller, Sophie Klusen, Stefan Paepke, Marion B. Kiechle, Daniela Paepke

**Affiliations:** 1 Department of Radiation Oncology, Klinikum Rechts der Isar, Medical School, Technical University Munich, 81675 Munich, Germany; Johanne.kleinevennekate@live.de (J.K.V.); Marciana.duma@tum.de (M.N.D.); maximilian.loos@yandex.com (M.L.); stephaniecombs@tum.de (S.E.C.); kilian.schiller@mri.tum.de (K.S.); sophie.klusen@mri.tum.de (S.K.); 2 ARCIM Institute (Academic Research in Complementary and Integrative Medicine), 70794 Filderstadt, Germany; j.vagedes@arcim-institute.de (J.V.); m.islam@arcim-institute.de (M.O.A.I.); 3 Department of Neonatology, University Children’s Hospital Tubingen, 72016 Tubingen, Germany; 4 Deutsches Konsortium für Translationale Krebsforschung (DKTK)-Partner Site Munich, 81675 Munich, Germany; 5 Helmholtzzentrum München, Institute of Radiation Medicine, 85764 Neuherberg, Germany; 6 Department of Gynecology and Obstetrics, Technical University of Munich (TUM), Ismaninger Strasse 22, 81675 Munich, Germany; Stefan.Paepke@mri.tum.de (S.P.); Marionkiechle@mri.tum.de (M.B.K.); Daniela.paepke@mri.tum.de (D.P.)

**Keywords:** breast cancer, radiotherapy, hypofractionation, organ-sparing, radiodermatitis

## Abstract

**Simple Summary:**

Moderate hypofractionated radiotherapy (HF) has become the standard fractionation scheme for most breast cancer patients. Despite comprehensive data from large, randomized trials, standardized assessment of patient reported outcome (PRO) and physiological changes after HF are largely missing. In this prospective trial focusing on radiodermatitis, HF and conventional normofractionated radiotherapy (CF) were compared using standardized Skindex-16 questionnaire in addition to CTCAE assessment and ultrasound measurement of the skin. The results of the current study complement and confirm existing evidence that HF leads to a lower degree of acute radiodermatitis and better patient reported outcome compared to CF.

**Abstract:**

The current study aims to determine whether hypofractionated radiotherapy (HF) leads to lower rates of acute radiodermatitis compared to conventional normofractionated radiotherapy (CF). A total of 166 patients with invasive breast cancer or DCIS were included in a prospective cohort study. Evaluation of acute radiodermatitis was obtained before radiotherapy, at the end of the treatment (T1), and 6 weeks after the treatment (T2) using CTCAE (v5.0) scores, the Skindex-16 questionnaire, and ultrasound measurement of the skin. CTCAE and Skindex-16 scores in the CF-group were significantly higher compared to the HF group indicating more pronounced side effects at the end of the treatment (CTCAE: CF-RT 1.0 (IQR: 0.0) vs. HF-RT 0.0 (0.25); *p* = 0.03; Skindex-16: CF: 20.8 (IQR: 25.8); HF: 8.3 (27.1); *p* = 0.04). At 6 weeks after the treatment, no significant differences between the two fractionation schemes were observed. Ultrasound based assessment showed that the skin thickness in the treated breast was higher compared to the healthy breast at all time-points. However, no significant difference between HF and CF was seen either at T1 or T2. The current study complements and confirms pre-existing evidence that HF leads to a lower degree of acute radiodermatitis and better patient reported outcome compared to CF at the end of treatment. This should be considered whenever fractionation of adjuvant breast cancer treatment is being discussed.

## 1. Introduction

Adjuvant radiotherapy in breast cancer patients effectively reduces the risk of locoregional recurrences and prolongs breast cancer specific survival [1]. In recent years, modern organ-sparing techniques such as deep-inspiration breath hold have been implemented in clinical practice to minimize the risk of late toxicities including cardiovascular diseases and secondary malignancies [2]. Despite these advances in prevention of late toxicity, the understanding and management of acute toxicity has remained unsatisfactory. Acute radiodermatitis is by far the most common acute side effect of adjuvant breast cancer radiotherapy, affecting 74–100% of all patients. Even though radiodermatitis represents a reversible and non-dose-limiting side effect, related complaints such as pain, itching, and burning have been shown to significantly reduce the quality of life during and after the treatment [3]. Furthermore, the extent of radiodermatitis determines how stressful a patient experiences the therapy [4].

Based on the START (Standardization of Breast Radiotherapy) B and Ontario Trial, moderate hypofractionated (HF) radiotherapy with 40–42.5 Gy in 15–16 Fx has become the standard radiotherapy regimen for adjuvant breast cancer. Both studies demonstrated oncological equivalence of conventional radiotherapy (CF) and HF as well as a reduction in acute (and late) skin toxicity for HF [5,6]. These results were confirmed by recently published 9-year follow-up data of a randomized Danish study (DBCG-HYPO trial [7]). Smaller prospective and retrospective studies focusing specifically on acute skin toxicity during hypofractionated radiotherapy consistently found that HF yields lower rates of acute skin toxicity compared to CF [8,9].

However, the assessment of skin toxicity in previous studies was predominantly based on clinical assessment by radiation oncologists. Most utilized scales were very brief and focused exclusively on physical reactions such as erythema, edema, and desquamation [10]. Standardized classification systems (CTCAE, RTOG) based on such physical parameters do not reliably reflect the patient’s burden and are characterized by considerable interobserver variability [11,12]. Therefore, more detailed assessment of patient-reported complaints and objective measurements indicating inflammatory changes are necessary to reliably obtain differences between CF-whole breast irradiation (WBI) and HF-WBI.

For subjective assessment of skin toxicity, an accurate and sensitive questionnaire (Skindex-16) has been developed by Chen et al. [11], which has been tested successfully in earlier studies on radiodermatitis [13]. Liu et al. [11] established, in addition to this, a quantitative and objective method to assess radiation-induced skin changes using ultrasonic imaging. According to their results, measuring the thickening of dermis post-radiation using B-mode images indicates inflammation and correlates with pathophysiological changes. Thus, ultrasound may be a suitable technique for quantification of acute and late toxicity in breast cancer patients undergoing radiotherapy.

Using these additional patient-related and objective measures, we aim to provide a comprehensive comparison of differences regarding radiation-induced skin changes between HF and CF. Additional analyses on this topic is warranted, as pattern of care analyses reveal persisting concerns regarding the acute toxicity of radiation hypofractionation even though it is considered the standard fractionation scheme for most breast cancer patients [14].

## 2. Materials and Methods

A total of 166 patients with invasive breast cancer or DCIS treated in our institute between December 2016 and May 2019 were enrolled in this prospective cohort study. Inclusion criteria comprised (1) adjuvant radiotherapy after breast conserving therapy (BET) or mastectomy and (2) age > 18 years. All patients gave informed consent for radiotherapy and participation in this trial. Treatment was carried out according to national guidelines and institutional standard operating procedures. Planning computer tomography (CT) scan of the thorax was obtained on a Somatom Emotion 16 scanner (Siemens Healthineers, Erlangen, Germany). Treatment planning was performed using Eclipse 15.6 (Varian Medical Systems, Palo Alto, CA, USA) and all treatment plans were approved in our center by a board of attending radiation oncologist prior to treatment. Prescribed dose was either 50.4 Gy in 28 Fx, 5 Fx per week, (*n* = 105) or 40.05 Gy in 15 Fx, 5 Fx per week, (*n* = 61) depending on date of treatment, with conventional normofractionated radiotherapy (CF) being the preferred schedule after lumpectomy for all patients treated before 2018. Patients undergoing postmastectomy radiotherapy and/or irradiation of the lymph node areas were predominantly treated with CF, irrespective of the date of treatment. All patients were treated with photons (6/10/15 MeV) except for one patient that received an electron boost. No bolus was used during the treatment. The EQD2 (assuming an α/β of 3.9 Gy [15]) was 48.7 Gy for CF and 44.6 Gy for HF. Patients receiving irradiation after lumpectomy without inclusion of regional lymph node areas were predominantly treated with 3D conformal radiotherapy, whereas patients receiving chest wall irradiation and/or irradiation of the lymph node areas were predominantly treated with IMRT/VMAT. Hierarchical clustering was used to define two subgroups receiving either CF (*n* = 38) or HF (*n* = 42) radiotherapy with similar risk factors for radiodermatitis. The risk factors considered for clustering were derived from previous studies [10,16,17] and comprised diabetes mellitus, cup size, chemo, smoking status, and application of topic cremes. Before the start of radiotherapy, all patients were offered the option of using Bepanthol lotion throughout the treatment. In case of symptomatic skin irritation ≥ CTCAE grade I, Bepanthen lotion was explicitly recommended. The use of Anthyllis gel was recommended to patients that were also treated in the Department of Integrative Medicine (Ingredients: Anthyllis vulneraria D3 tincture 10 g, hydroxyethyl cellulose 30 g). Patient characteristics of both groups are summarized in Table 1.

For the final patient collective of this study, evaluation of the treatment plans including PTV_Dmax, PTV_V80%, and PTV_V107% was performed [16]. Evaluation of acute radiodermatitis was obtained before radiotherapy, at the end of the treatment, and 6 weeks after the treatment. The CTCAE (v5.0) score was obtained by an experienced radiation oncologist at all time points. In addition, patients were asked to answer the Skindex-16 questionnaire [11], which addresses patients’ health-related quality of life and the psychosocial and physical effects due to skin toxicity by measuring patient’s emotions (e.g., fear, shame, and anger), symptoms (e.g., itching and pain), and functions (e.g., daily activities and social life). For objective assessment of the skin changes during the radiotherapy, a specialist for breast ultrasound (German Society of Ultrasound in Medicine (DEGUM) level 2) performed scans of the treated breast before, at the end, and 6 weeks after the treatment. The skin thickness was measured with 18-4bMHz ultrasonic probe using a Philips EPIQ 7w with a linear transducer. The patient was laid in a supine position on the examination bed with both arms up, behind the head. The thickness of the skin was measured at four different locations at each breast: 12:00, 3:00, 6:00, and 9:00 o’clock around the mamilla. The position of the transducer was kept vertical to the surface and was carefully placed on the skin with minor pressure to avoid affecting the measurements of the skin. All images were printed and attached to the ultrasound protocol where skin thickness was quantified in millimeters.

Clustering and statistical analyses were performed using the programming language R (version 4.0.2) running in RStudio (version 1.2.5033). Hierarchical clustering method to obtain clusters of similar sub-populations as described above was performed using the package factoextra. The baseline values (demographic data) from our study, are reported descriptively, i.e., absolute and relative frequency for categorical or binary variables, median (interquartile range (IQR)) for continuous variables. All parameters in the paper were not normally distributed. Therefore, differences between the two subgroups were tested for statistical significance using the Wilcoxon signed-rank test.

## 3. Results

### 3.1. Dose Distribution PTV

Dose distribution in the PTV was similar between the CF-RT and HF-RT group: PTV_Dmax: 107.2% vs. 107.0% (*p* = 0.72); PTV_V80%: 93.0% vs. 90.2% (*p* = 0.08); and PTV_V107%: 0.06% vs. 0.02% (*p* = 0.42).

### 3.2. Differences between CTCAE Scores

**Figure 1:**
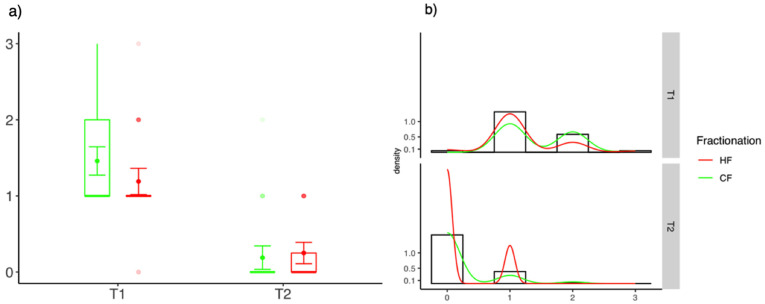
Differences of CTCAE scores between HF-WBI and CF-RT at the end of radiotherapy (T1) and 6 weeks after the treatment (T2). (**a**) Box-Plot diagrams and (**b**) distribution of values.

At the end of the treatment (T1) CTCAE scores in the CF-group were significantly higher compared to the HF group (CF-RT 1.0 (0.0) vs. HF-RT 0.0 (0.25); *p* = 0.03). Six weeks after the treatment (T2), only small and insignificant differences between the two groups were observed (CF-RT 0.0 (0.0) vs. HF-RT 0.0 (0.3); *p* = 0.39). Figure 1 delineates the differences of CTCAEs scores at the end of treatment and at 6 weeks follow-up.

### 3.3. Differences between Skindex-16

The Skindex-16 scores at the end of radiotherapy were significantly higher compared to the baseline assessment indicating an impairment of emotions, symptoms, and/or functioning caused by the treatment. The Skindex-16 scores were significantly higher after CF compared to HF. Six weeks after the treatment, the Skindex-16 scores were similar to the baseline scores and no significant difference between CF and HF was observed. The results are summarized in Table 2.

### 3.4. Differences of Skin Thickness

**Figure 2:**
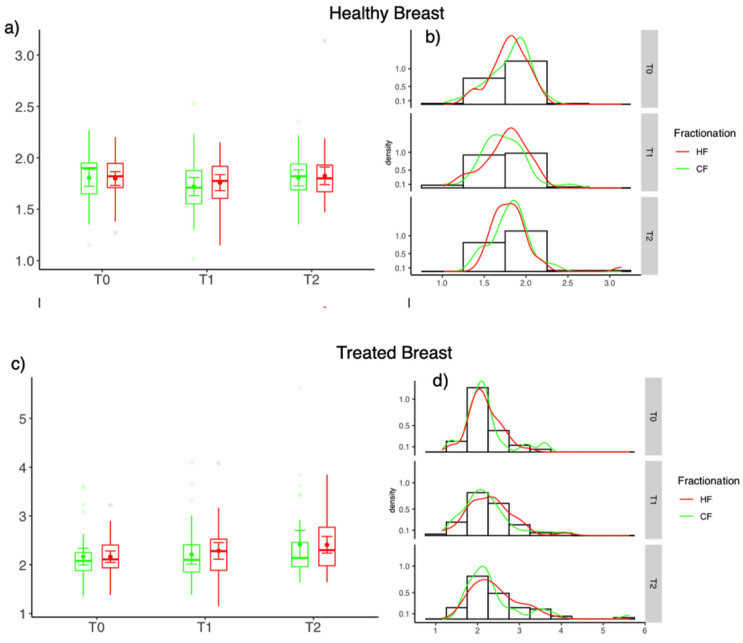
Differences of skin thickness between HF (red) and CF (green) at the end of radiotherapy (T1) and 6 weeks after the treatment (T2). Healthy breast (**a**,**b**) and treated breast (**c**,**d**); (**a**,**c**) Box-Plot diagrams and (**b**,**d**) distribution of values.

The skin thickness measured by ultrasound was higher compared to the healthy breast at all time-points. For the treated breast the skin thickness increased after the treatment. However, no significant difference between HF-WBI and CF-WBI was seen both at T1 and T2 (Figure 2).

## 4. Discussion

The current prospective evaluation of physician- and patient-reported outcomes confirms that hypofractionated whole breast irradiation yields lower rates of acute toxic effects compared to normofractionated radiotherapy. The objective assessment of skin changes based on ultrasound did not indicate significant differences between HF and CF.

One of the main reasons national and international guidelines consistently adopted hypofractionation after the publication of the randomized START B and Ontario Trial was the favorable toxicity profile of this dose regimen. Nonetheless, doubts about increased toxicity persist [14], especially due to a lack of patient-related outcome parameters, as well as objective measurement criteria in previous studies. Based on a review by Schnur et al. [10] only 9% of the studies on radiodermatitis included a patient-rated measure, and most studies focused almost exclusively on physical reactions.

A strength of the current study is the detailed assessment of patients reported outcomes. No significant difference of “symptoms” was reported between the CF and HF group at the end of the treatment based on the Skindex-16 questionnaire, despite clear differences in CTCAE scores. Instead, we observed a significant difference for “emotions” based on the appearance of the skin. This emphasizes the importance of evaluation of patient reported outcomes, as physicians tend to focus on the symptoms of radiodermatitis (itching, burning, and pain), instead of the cosmetic and emotional impact [10].

Our results indicate a favorable impact of moderate HF based on patient and physician assessment in accordance with earlier studies on the subject. A randomized study by Shaitelman et al. [18] compared acute side effects measured by CTCAE scores and the FACT-B questionnaire between hypofractionation and normofractionated radiotherapy, and showed significantly higher rates of maximum physician-reported acute dermatitis (36% vs. 69%; *p* < 0.001), pruritus (54% vs. 81%; *p* < 0.001), breast pain (55% vs. 74%; *p* = 0.001), hyperpigmentation (9% vs. 20%; *p* = 0.002), and fatigue (9% vs. 17%; *p* = 0.02) in patients receiving normofractionated radiotherapy. For patient reported outcome, no data were evaluated immediately after the end of radiotherapy. At 6-month follow-up, outcomes for “lack of energy” and “trouble meeting family needs” favored patients randomized to HF. A previous analysis of Quality of Life (QOL) outcomes from the START trials revealed a lower rate of patient-reported moderate to marked breast, arm, and shoulder symptoms in patients randomized to HF [19]. Once again, acute toxicity immediately after the treatment was not reported. In our current study, we focused on side effects immediately after treatment and within the first 6 weeks, complementing the existing evidence on skin toxicity after hypofractionated radiotherapy.

Summarizing the available evidence, hypofractionated radiotherapy seems to be superior compared to CF with regard to acute radiodermatitis. The improvement of quality of life and the lower degree of skin toxicity during hypofractionation radiotherapy should be taken into consideration whenever the optimal fraction scheme is being discussed (e.g., DCIS, Postmastectomy irradiation, or lymph node irradiation).

The objective assessment of skin thickness by ultrasound did not show a significant difference between hypofractionated radiotherapy and normofractionated radiotherapy. This might be attributable to the fact that most patients developed only mild radiodermatitis and differences between HF and CF were too small to be detected by ultrasound. However, the treated breast clearly showed a larger skin thickness compared to the healthy contralateral side, which is in accordance with earlier studies [20].

In a randomized trial, Schmeel at al. [8] observed a decreased erythema severity and hyperpigmentation in the hypofractionation arm compared to CF using spectrophotometry. Spectrophotometry can reliably detect and quantify erythema, which is frequently the first reaction of the irradiated skin. However, it cannot detect changes in the deeper layers of the skin, which can be particularly relevant for the long-term effects. Hence, the optimal method for objective and reproducible assessment of radiotherapy remains unclear.

In the START B and Ontario trials, a single dose of 2 Gy was used for CF. However, according to pattern-of-care surveys, a schedule of 50.4 Gy in 28 Fx is more commonly used during CF in daily practice [14], which is why this schedule was chosen for the current study. Both fractionation schedules are within the recommended range for CF in NCCN guidelines [21] and differ only slightly with regard to EQD2 and total treatment time.

A potential limitation of this current trial is the fact that the course of the radiodermatitis was not monitored until the first follow-up after 6 weeks. Therefore, it is possible that in some patients the “peak” of radiation dermatitis was not detected.

## 5. Conclusions

Hypofractionation leads to a lower degree of acute radiodermatitis compared to CF and is associated with better patient reported outcome in adjuvant breast cancer radiotherapy. This should be taken into consideration whenever HF and CF are being discussed as potential treatment options. Further, our results emphasize the importance of patient reported outcomes for the correct assessment of radiodermatitis in future trials (e.g., on ultra-hypofractionation), as CTCAE scores and objects measurements do not reliably reflect the patient’s perception.

## Figures and Tables

**Table 1:** Patients’ characteristics after hierarchical clustering. Missing values account for deviations from 100% in same categories.

Variable		CF	HF	Total
	*n* (%)	38 (47.5%)	42 (52.5%)	80 (100.0%)
Age				
	Mean (SD)	55.6 (11.9)	61.3 (13.0)	58.6 (12.8)
Body Mass Index				
	Mean (SD)	25.1 (3.8)	24.7 (4.5)	24.9 (4.2)
Diabetes Mellitus				
	No	37 (97.4%)	42 (100.0%)	79 (98.8%)
Smoker				
	No	36 (94.8%)	42 (100.0%)	78 (97.5%)
Cup-Size				
	A	1 (2.6%)	8 (19.1%)	9 (11.3%)
	B	19 (50.0%)	13 (31.0.%)	32 (40.0%)
	C	10 (26.3%)	10 (23.8%)	20 (25.0%)
	D	4 (10.5%)	7 (16.7%)	11 (13.8%)
	E	1 (2.6%)	1 (2.4%)	2 (2.5%)
	F	0 (0.0%)	1 (2.4%)	1 (1.3%)
Side				
	Right	25 (65.8%)	23 (54.8%)	48 (60.0%)
	Left	13 (34.2%)	19 (45.3%)	32 (40.0%)
Topic Cremes				
	Bepanthen	16 (42.1%)	16 (38.1%)	32 (40.0%)
	No Creme	15 (39.5%)	18 (42.9%)	33 (41.3%)
	Anthyllis	4 (10.5%)	4 (9.5%)	8 (10.0%)
	Both	3 (7.9%)	4 (9.5%)	7 (8.8%)
Surgery Typ				
	BET	30 (79.0%)	41 (97.6%)	71 (88.8%)
	Mastectomy	7 (18.4%)	1 (2.4%)	8 (10.0%)
	others	1 (2.6%)	0 (0.0%)	1 (1.3%)
Lymph Node surgery				
	SLNB	16 (42.1%)	39 (92.9%)	55 (68.8%)
	None	12 (31.6%)	0 (0.0%)	12 (15.0%)
	ALND	10 (26.3%)	3 (7.1%)	13 (16.3%)
Chemotherapy				
	None	29 (76.3%)	38 (90.5%)	67 (83.8%)
	Neo-Adjuvant	8 (21.1%)	4 (9.5%)	12 (15.0%)
	Adjuvant	1 (2.6%)	0 (0.0%)	1 (1.3%)
Lymph node irradiation				
	0	24 (63.2%)	40 (95.2%)	64 (80.0%)
	1	14 (36.8%)	1 (2.4%)	15 (18.8%)
Photon Energy				
	6 MV	22 (57.9%)	21 (50.0%)	43 (53.8%)
	6/15 MV	12 (31.6%)	15 (35.7%)	27 (33.8%)
	6/10 MV	4 (10.53%)	1 (2.38%)	5 (6.25%)
Boost				
	Yes	20 (52.6%)	29 (69.1%)	49 (61.3%)
	No	18 (47.4%)	13 (31.0%)	31 (38.8%)

**Table 2:** Differences of CF and HF based on Skindex-16 questionnaire for three different categories (emotions, symptoms, and functioning). Missing values account for deviations from 100% in same categories.

Variable	T	CF	HF	CF vs. HF
Emotions				
	T0	0 (8.9)	0 (4.8)	0 (0; 0) *p* = 0.68
	T1	19.1 (25.0)	7.1 (27.4)	11.9 (0; 14.3) *p* = 0.05
	T2	0.0 (7.1)	0 (13.1)	0.0 (0; 0.0) *p* = 0.76
Symptoms				
	T0	0 (12.5)	4.2 (12.5)	−4.2 (0; 0) *p* = 0.79
	T1	33.3 (41.7)	29.2 (41.7)	4.2 (−0.0; 20.8) *p* = 0.11
	T2	8.3 (17.7)	4.17 (25.0)	4.2 (0; 4.2) *p* = 0.55
Functioning				
	T0	0 (6.67)	0 (0.0)	0 (0; 0) *p* = 0.13
	T1	6.7 (16.67)	0 (10.0)	6.7 (0; 6.7) *p* = 0.15
	T2	0 (6.7)	0 (3.3)	0 (0; 0) *p* = 0.57
Total				
	T0	1.0 (8.3)	2.1 (6.3)	−1.0 (−1.0; 1.04) *p* = 0.84
	T1	20.8 (25.8)	8.3 (27.1)	12.5 (−1.0; 14.6) *p* = 0.04
	T2	3.7 (7.6)	2.1 (9.6)	1.6 (−2.1; 3.1) *p* = 0.55

## Data Availability

The data presented in this study are available on request from the corresponding author. The data are not publicly available due to ethical reasons.

## References

[1] Darby S., McGale P., Correa C., Taylor C., Arriagada R., Clarke M., Cutter D., Davies C., Ewertz M., Early Breast Cancer Trialists’ Collaborative Group (2011). Effect of radiotherapy after breast-conserving surgery on 10-year recurrence and 15-year breast cancer death: Meta-analysis of individual patient data for 10,801 women in 17 randomised trials. Lancet.

[2] Duma M.N., Baumann R., Budach W., Dunst J., Feyer P., Fietkau R., Haase W., Harms W., Hehr T., Krug D. (2019). Heart-sparing radiotherapy techniques in breast cancer patients: A recommendation of the breast cancer expert panel of the German society of radiation oncology (DEGRO). Strahlenther Onkol..

[3] López E., Núñez M.I., Guerrero M.R., Del Moral R., Luna J.D.D., Rodríguez M.D.M., Valenzuela M.T., Villalobos M., De Almodóvar J.M.R. (2002). Breast cancer acute radiotherapy morbidity evaluated by different scoring systems. Breast Cancer Res. Treat..

[4] Schnur J.B., Ouellette S.C., DiLorenzo T.A., Green S., Montgomery G.H. (2011). A qualitative analysis of acute skin toxicity among breast cancer radiotherapy patients. Psychooncology.

[5] Arsenault J., Parpia S., Goldberg M., Rakovitch E., Reiter H., Doherty M., Lukka H., Sussman J., Wright J., Julian J. (2020). Acute Toxicity and Quality of Life of Hypofractionated Radiation Therapy for Breast Cancer. Int. J. Radiat. Oncol. Biol. Phys..

[6] Bentzen S.M., Agrawal R.K., Aird E.G., Barrett J.M., Barrett-Lee P.J., Bliss J.M., Brown J., Dewar J.A., Dobbs H.J., START Trialists’ Group (2008). The UK Standardisation of Breast Radiotherapy (START) Trial A of radiotherapy hypofractionation for treatment of early breast cancer: A randomised trial. Lancet Oncol..

[7] Offersen B.V., Alsner J., Nielsen H.M., Jakobsen E.H., Nielsen M.H., Krause M., Stenbygaard L., Mjaaland I., Schreiber A., Kasti U.-M. (2020). Hypofractionated Versus Standard Fractionated Radiotherapy in Patients With Early Breast Cancer or Ductal Carcinoma In Situ in a Randomized Phase III Trial: The DBCG HYPO Trial. J. Clin. Oncol..

[8] Schmeel L.C., Koch D., Schmeel F.C., Röhner F., Schoroth F., Bücheler B.M., Mahlmann B., Leitzen C., Schüller H., Tschirner S. (2020). Acute radiation-induced skin toxicity in hypofractionated vs. conventional whole-breast irradiation: An objective, randomized multicenter assessment using spectrophotometry. Radiother. Oncol..

[9] Tortorelli G., Di Murro L., Barbarino R., Cicchetti S., Di Cristino D., Falco M.D., Fedele D., Ingrosso G., Janniello D., Morelli P. (2013). Standard or hypofractionated radiotherapy in the postoperative treatment of breast cancer: A retrospective analysis of acute skin toxicity and dose inhomogeneities. BMC Cancer.

[10] Schnur J.B., Love B., Scheckner B.L., Green S., Gabriella A., Montgomery G.H. (2011). A systematic review of patient-rated measures of radiodermatitis in breast cancer radiotherapy. Am. J. Clin. Oncol..

[11] Chren M.M., Lasek R.J., Sahay A.P., Sands L.P. (2001). Measurement properties of Skindex-16: A brief quality-of-life measure for patients with skin diseases. J. Cutan. Med. Surg..

[12] De Langhe S., Mulliez T., Veldeman L., Remouchamps V., Van Greveling A., Gilsoul M., De Schepper E., De Ruyck K., De Neve W., Thierens H. (2014). Factors modifying the risk for developing acute skin toxicity after whole-breast intensity modulated radiotherapy. BMC Cancer.

[13] Rzepecki A., Birnbaum M., Ohri N., Daily J., Fox J., Bodner W., Kabarriti R., Garg M., Mehta K., Kalnicki S. (2019). Characterizing the Effects of Radiation Dermatitis on Quality of Life: A Prospective Survey-Based Study. J. Am. Acad. Dermatol..

[14] Mayinger M., Straube C., Habermehl D., Duma M., Combs S. (2020). Hypo- vs. normofractionated radiation therapy in breast cancer: A patterns of care analysis in German speaking countries. Rep. Pract. Oncol. Radiother..

[15] Qi X.S., White J., Li X.A. (2011). Is alpha/beta for breast cancer really low?. Radiother. Oncol..

[16] Borm K.J., Loos M., Oeschsner M., Mayinger M., Paepke D., Kiechle M.B., Combs S.E., Duma M.N. (2018). Acute radiodermatitis in modern adjuvant 3D conformal radiotherapy for breast cancer–The impact of dose distribution and patient related factors. Radiat. Oncol..

[17] Chen M.-F., Chen W.-C., Lai C.-H., Hung C.-H., Liu K.-C., Cheng Y.-H. (2010). Predictive factors of radiation-induced skin toxicity in breast cancer patients. BMC Cancer.

[18] Shaitelman S.F., Schlembach P.J., Arzu I., Ballo M.T., Bloom E.S., Buchholz D., Chronowski G.M., Dvorak T., Grade E., Hoffman K.E. (2015). Acute and Short-term Toxic Effects of Conventionally Fractionated vs Hypofractionated Whole-Breast Irradiation: A Randomized Clinical Trial. JAMA Oncol..

[19] Hopwood P., Haviland J., Sumo G., Mills J., Bliss J., Yarnold J.R. (2010). Comparison of patient-reported breast, arm, and shoulder symptoms and body image after radiotherapy for early breast cancer: 5-year follow-up in the randomised Standardisation of Breast Radiotherapy (START) trials. Lancet Oncol..

[20] Liu T., Zhou J., Osterman K.S., Zhang P., Woodhouse S.A., Schiff P.B., Kutcher G.J. (2008). Measurements of Radiation-Induced Skin Changes in Breast-Cancer Radiation Therapy Using Ultrasonic Imaging. Annu. Int. Conf. IEEE Eng. Med. Biol. Soc..

[21] National Comprehensive Cancer Network (2018). Breast Cancer- Version 8.2021. https://www.nccn.org/professionals/physician_gls/pdf/breast.pdf.

